# Validation of the Relational Self-Esteem Scale in Ecuador: Unidimensional Structure and Gender Invariance

**DOI:** 10.11621/pir.2026.0102

**Published:** 2026-03-01

**Authors:** Aitor Larzabal-Fernandez, Marlon Mayorga-Lascano, Diana Carolina Validiviezo Rodríguez, Giovanni Lascano-Arias, Rocío Játiva Morillo, Carlos Rodrigo Moreta-Herrera

**Affiliations:** a *University of the Basque Country - EHU (UPV/EHU), Bilbao, Spain*; b *Pontifical Catholic University of Ecuador (PUCE), Ambato, Ecuador*; c *Technical University of Madrid, Spain*; d *Technological University Indoamérica, Ambato, Ecuador*; e International University SEK, Quito, Ecuador

**Keywords:** relational self-esteem, Ecuador, self-esteem, invariance, gender, confirmatory factor analysis

## Abstract

**Background:**

Relational self-esteem — self-worth derived from significant relationships — is central to psychosocial functioning. However, brief measures validated with appropriate methods for ordinal items are scarce in Ecuadorian populations.

**Objective:**

To examine the factorial structure, internal consistency, convergent validity, and gender-based measurement invariance of the Relational Self-Esteem Scale (RSE), using a cross-validation approach.

**Design:**

Instrumental, cross-sectional study with *n* = 1.478 Ecuadorian adults. The sample was split for cross-validation: 40% (*n =* 591) for EFA and 60% (*n =* 887) for CFA. EFA used a polychoric matrix, ULS extraction, and oblimin rotation; factor retention was guided by parallel analysis. CFA employed WLSMV. Convergent validity was tested against personal self-esteem (RSES), collective self-esteem (CSE), and mental well-being (MHC-SF). Multigroup measurement invariance by gender was assessed (configural, metric, scalar, strict).

**Results:**

Parallel analysis and confirmatory factor analysis supported a unidimensional structure with robust factor loadings and an adequate overall model fit. The scale demonstrated high internal consistency and reliability. Convergent validity was established through significant positive correlations with measures of self-esteem, core self-evaluations, and mental health. The instrument showed full measurement invariance across genders, meeting all criteria from configural to strict levels.

**Conclusion:**

The RSE shows a unidimensional structure, adequate reliability, convergent validity, and gender invariance in Ecuadorian adults. Findings support using the total score as a brief, valid indicator of relational self-esteem in this context.

## Introduction

Self-esteem is a central component of psychological functioning and subjective well-being. Traditionally, it has been defined as the global and affective evaluation that one makes of oneself (Rosenberg, 1965). Longitudinal research has shown that high self-esteem prospectively predicts success and well-being in fundamental areas such as relationships, work, and health (Orth & Robins, 2014). A recent quantitative synthesis of 40 meta-analyses (Zell & Johansson, 2024) evidenced a solid relationship between self-esteem and well-being, consistent across different cultural regions. Likewise, self-esteem has been identified as a protective factor that buffers the effects of stress and promotes psychological resilience (Tang et al., 2024). The role of self-esteem can also be conditioned by contextual factors, through a relational and dynamic process, dependent on social interaction and symbolic ties with others (Jiang, 2020).

However, the conception of the self is not universal; there are different forms of self-definition depending on the cultural context: the “independent self,” centered on internal and unique attributes, and the “interdependent self,” which emphasizes connection and relationships with others (Markus & Kitayama, 1991). From this perspective, sources of self-evaluation may go beyond the individual, including relational and collective dimensions. Brewer and Gardner (1996) proposed a tripartite model of the self — personal, relational, and collective — that integrates different forms of self-representation with differentiated origins and sources of self-evaluation.

Based on this approach, Luhtanen and Crocker (1992) developed the Collective Self-Esteem Scale, and later Du, King, and Chi (2012) introduced the Relational Self-Esteem Scale (RSE), defining relational self-esteem as the positive evaluation of oneself derived from meaningful relationships with other people. This approach is aligned with the notion of relational–interdependent self-construal (Cross, Bacon, & Morris, 2000) and with the relational levels of self-definition described by Brewer and Gardner (1996).

Since its development, the RSE has demonstrated predictive relevance, being positively associated with life satisfaction (Du, King, & Chi, 2017) and negatively with perceived stress (Tang et al., 2024). Nevertheless, an analysis of its psychometric evidence reveals two crucial issues: ambiguity in its factor structure and a marked geographical concentration of studies in the Asian context (Du et al., 2012; Du et al., 2016). Although its reliability is consistently adequate, with Cronbach’s alpha coefficients above .70 (Du et al., 2016; Li et al., 2022), its internal structure is inconsistent. The original study supported a one-dimensional model in university students (Du et al., 2012), whereas a later study found that a two-factor model (family and friends) fit better in a rural community sample (Du et al., 2016). Finally, regarding measurement invariance, the researchers confirmed factorial invariance of the scale between a clinical sample (HIV-positive caregivers) and a non-clinical sample (HIV-negative caregivers) (Du et al., 2016).

The literature review reflects the broad application of this scale across various studies. Beyond the predictive associations mentioned above, research has also linked the instrument to constructs such as happiness and positive affect (Du et al., 2017), as well as personal self-esteem, resilience, and social support (Du et al., 2016), among others. In addition, relational self-esteem has shown moderation by gender in gender stereotypes in men (Li, Liu, & Song, 2022); however, there is a risk that the relationship between items and construct is not equivalent in men and women, since there are no studies that analyze gender invariance. It is generally assumed that scales measure constructs in the same way across different groups (Byrne, 2006); however, differences may be due to the instrument having different factorial solutions depending on the comparison group (Asparouhov & Muthén, 2014). Therefore, it is important to analyze invariance by group to avoid this type of error.

Despite the growing international evidence, the measurement of relational self-esteem in Latin American contexts is still limited (Barreda Parra, 2021; Diori, 2018; Yomtov et al., 2015). The evidence on the psychometric properties of the tool has been mainly in the Asian context (Du et al., 2012; Du, Li, et al., 2018), in which cultural conceptions of the “self” and relationships may differ substantially from Latin American cultural conceptions (Oyserman et al., 2002). In this regard, the need has been noted to adapt and validate measures that capture the particularities of relational experience in interdependent societies, where the sense of belonging and emotional reciprocity play a central role in the construction of personal identity.

In Ecuador, and in Latin America in general, family, community, and friendship relationships are fundamental sources of emotional support and self-esteem (Moreta-Herrera et al., 2025; Valle Pico, 2022). However, to date no psychometric evidence has been found regarding the validity and reliability of instruments that assess relational self-esteem in this context. Having an adapted and validated measure would allow progress in understanding the social and emotional predictors of well-being, as well as in the design of cultural and educational interventions aimed at strengthening interpersonal bonds.

The present study thus seeks to contribute to the development of culturally sensitive psychometric tools for the study of the relational self in Latin America, and to strengthen the understanding of the role that interpersonal relationships play in the construction of self-esteem and mental health in interdependent social contexts.

The study’s objective is to identify validity evidence from Classical Test Theory (CTT) for the RSE in a large Ecuadorian sample. It is hypothesized that the RSE presents an adequate internal structure (H1), as well as convergent validity with other measures of self-esteem and mental health (H2); that the RSE also has adequate reliability of internal consistency (H3); and that the RSE has properties of measurement equivalence based on gender (H4).

## Methods

The present study corresponds to a descriptive work of an instrumental nature or an analysis of psychometric properties (Ato et al., 2013), in which validity evidence of the RSE is analyzed in a sample of university students from Ecuador.

### Participants

The sample consisted of 1.478 people residing in Ecuador, selected through non-probability incidental sampling. Participants’ ages ranged from 18 to 78 years (*M* = 23.93; *SD =* 7.27), of whom 56.6% were women and 43.4% men. All participants voluntarily completed the online questionnaire, guaranteeing anonymity and confidentiality of responses.

### Instruments

*The Relational Self-Esteem Scale* (RSE) developed by Du et al. (2012) assesses the degree to which people experience a positive evaluation of themselves based on their meaningful interpersonal relationships. It consists of 7 items with a 7-point Likert-type response format (1 = Strongly disagree to 7 = Strongly agree), where higher scores reflect greater relational self-esteem. Example items: “I am a worthy member of my circle of friends,” “I think my family is proud of me.” Regarding internal consistency in the study, the values were excellent (α = .86, 95% CI [.85, .88]; ω = .86; 95% CI [.85, .87]). The Spanish version of the instrument was obtained through a back-translation process conducted in a prior international study. This involved translation by native bilingual speakers and a subsequent back-translation by a committee of psychologists to ensure conceptual and linguistic equivalence with the original version.

*The Rosenberg Self-Esteem Scale* (RSES; Rosenberg, 1965) in the Spanish version validated in Ecuador (Bueno-Pacheco et al., 2020). It is one of the most widely used instruments to assess global self-esteem. It consists of 10 items with a 4-point Likert format (1 = Strongly disagree to 4 = Strongly agree). Example item: “I feel that I have positive qualities.” Five items are worded negatively and require reverse scoring before computing the total score. The scale assesses a general factor of global self-esteem, understood as the affective and cognitive evaluation that one makes of oneself. Regarding reliability estimates, the following were obtained (α = .79, 95% CI[.76, .81]; ω = .78; 95% CI [.75, .80]).

*The Collective Self-Esteem Scale* (CES; Luhtanen & Crocker, 1992) measures the degree to which people positively value their belonging to social groups to which they belong. It is composed of 16 items, distributed in four subscales of 4 items each: Membership Collective Self-Esteem (*e.g*., “I am a worthy member of the groups I belong to”), Private Collective Self-Esteem (*e.g*., “I am proud of the groups I belong to”), Public Collective Self-Esteem (*e.g.*, “Overall, others think positively of the groupsI belong to”), and Importance to Identity (*e.g.*, “Belonging to my groups is an important part of my self-concept”). Responses are recorded on a 7-point Likert format (1 = Strongly disagree to 7 = Strongly agree). Regarding reliability, the results were as follows (α = .88, 95% CI [.86, .89]; ω = .83; 95% CI [.82, .84]).

*The Mental Health Continuum–Short Form* (MHC–SF; Keyes, 2002) in the Spanish version validated in the Ecuadorian context (Moreta-Herrera et al., 2025). The MHC-SF evaluates positive mental well- being through 14 items that measure three dimensions: a) Emotional well-being (*e.g.*, “During the past month, how often did you feel happy?”); b) Psychological well-being (*e.g*., “During the past month, how often did you feel that your life has a sense of meaning?”); and c) Social well-being (*e.g.*, “During the past month, how often did you feel that you belong to a community?”). Items are answered on a 6-point Likert scale (0 = Never to 5 = Every day). In this study, high reliability was obtained (α = .95, 95% CI [.94 – .95] and ω = .95, 95% CI [.94 – .95]).

### Procedure

Data were collected through an online survey designed in Qualtrics, within the framework of the Predictors of Mental Health in Ecuador Project, which is pre-registered in the Open Science Framework (OSF). Participation was voluntary, after reading and accepting the informed consent, approved by the corresponding institutional ethics committee (CEISH-UISEK-EX-EO-2024-002-2). No financial incentives were offered.

### Data Analysis

To ensure the robustness and replicability of the model, the total sample (*N =* 1.478) was randomly divided into two independent subsamples: 40% (*n =* 591) was used for the exploratory factor analysis (EFA) and the remaining 60% (*n =* 887) for the confirmatory factor analysis (CFA). This cross-validation strategy avoids overfitting and allows verifying the stability of the factor structure in an independent sample (Brown, 2015; Worthington & Whittaker, 2006).

1. Reliability: Cronbach’s α and McDonald’s ω coefficients were estimated with their respective 95% confidence intervals (95% CI), as well as item–total correlations and ω values if the item was deleted.
2. Exploratory factor analysis (EFA): Given the ordinal nature of the items, a polychoric correlation matrix was estimated and unweighted least squares (ULS) was used as the extraction method, due to its robustness to non-normality and its compatibility with polychoric matrices. The adequacy of the matrix was verified using the KMO index and Bartlett’s sphericity test, and factor retention was determined by parallel analysis.
3. Confirmatory factor analysis (CFA) was estimated using the WLSMV (Weighted Least Squares Mean and Variance Adjusted) method, due to the ordinal nature of the responses and the absence of multivariate normality. The fit indices evaluated were: chi-square (χ^2^), Comparative Fit Index (CFI), Tucker–Lewis Index (TLI), Root Mean Square Error of Approximation (RMSEA) with 90% confidence intervals (90% CI), and Standardized Root Mean Square Residual (SRMR), following the criteria of Hu and Bentler (1999).
4. Convergent validity was assessed through a matrix of Pearson correlations between the RSE and the other psychological measures (collective self-esteem, personal self-esteem, and mental health).
5. Multigroup factorial invariance was tested by gender through multigroup CFA (MG-CFA), following the configural, metric (loadings), scalar (intercepts), and strict (residuals) sequence, with WLSMV estimation. Invariance was evaluated based on changes in CFI(≤ .010) and RMSEA (≤ .015) between nested models (Cheung & Rensvold, 2002).

All analyses were performed in JASP 0.95.3 and JAMOVI 2.6.26, reporting 95% confidence intervals.

## Results

The reliability of the Relational Self-Esteem Scale (RSE) was high, with α = .869, 95% CI [.852 – .885] and ω = .869, 95% CI .859 – .879], indicating excellent internal consistency. Item–total correlations ranged from .61 to .77, and omega coefficients if the item was deleted remained high (.844 – .900), confirming the homogeneity of the construct (*Table 1*).

**Table 1:** Reliability Statistics of the Relational Self-Esteem Scale

**Item**	**ωid**	**αid**	**Itr**	**M**	**SD**	**Skewness**	**Kurtosis**
I am a worthy member of my circle of friends.	.890	.889	.702	4.637	1.922	–.372	–.846
I feel I have much to offer to my family.	.884	.883	.755	4.862	1.852	–.506	–.624
In general, I’m glad to be a member of my circle of friends.	.887	.885	.732	4.846	1.793	–.442	–.598
I am proud of my family	.894	.893	.666	4.781	1.709	–.378	–.378
Overall, my circle of friends is considered to be good by others.	.883	.881	.771	5.085	1.754	–.609	–.389
I think my family is proud of me.	.887	.885	.740	5.018	1.759	–.611	–.391
I can help my friends a lot.	.900	.898	.615	4.903	1.771	–.493	–.541

*Note. Ωid = Omega if item dropped; αid = alpha if item dropped; Itr=Item total correlation;M = Mean; SD = Standard Deviation*

The EFA was performed on the first subsample (40%, *n =* 591) with a matrix of polychoric correlations. It began with the test of sampling adequacy, KMO = .883, and Bartlett’s test of sphericity χ^2^(21) = 1431.80, *p <* .001, which indicated the relevance of the analysis. In addition, parallel analysis suggested a unifactorial structure, with a single component with an eigenvalue of 3.26, which explained 47.8% of the total variance. Factor loadings (λ) ranged from .64 (*item 7*) to .82 (*item 5*), all exceeding the minimum recommended threshold of λ > .40.

With respect to the CFA, the one-dimensional model proposed by the EFA was tested in the second subsample (60%, *n =* 887) through CFA with WLSMV estimation. This confirmed the model, showing adequate fit indices: χ^2^(11) = 70.25, *p <* .001; CFI= .990; TLI = .981; RMSEA = .077 [90% CI .060–.094]; SRMR = .021. Likewise, all item factor loadings were significant (*p <* .001) and ranged from .59 (*item 7*) to .75 (*item 5*). Moreover, item *R*^2^values ranged from .38 (*item 7*) to .55 (*item 2*). Finally, composite reliability was ω = .817 and α = .870, confirming the internal stability of the model.

In addition, modification indices were inspected. Three theoretically justified residual covariances were retained: (a) AUTREL1 ↔ AUTREL2, due to semantic overlap in belonging/affect within the friends referent; (b) AUTREL6 ↔ AUTREL7, for sharing a common source of evaluation (hetero-evaluation); and (c) AUTREL4 ↔ AUTREL5, for a prosocial style/positive valuation of the group and one’s own contribution. These covariances were kept equal by gender in the multigroup models (or freed if necessary), and their inclusion improved fit without altering the one-factor structure. As a sensitivity analysis, the same model was estimated without residual covariances; fit decreased (ΔCFI/ΔRMSEA), supporting the local need for these correlations by content and method.

Regarding convergent validity (*Table 2*), the correlation of the RSE with the RSES (*r =* .201, *p <* .001) and with the CES (*r =* .421, *p <* .001) shows significant and positive relationship with conceptually related measures. The MHC correlated positively and significantly with the RSE (*r =* .125, *p <* .001), RSES (*r =* .462, *p <* .001), and CES (*r =* .236, *p <* .001).

**Table 2:** Bivariate Correlations and Confidence Intervals among the RSE, RSES, CES, and MHC-SF

		**1**	**2**	**3**	**4**
	Pearson’s R	—			
1. RSE	Higher 95% CI	—			
	Lower 95% CI	—			
	Pearson’s R	.20***	—		
2. RSES	Higher 95% CI	.25	—		
	Lower 95% CI	.15	—		
	Pearson’s R	.42***	.02	—	
3. CES	Higher 95% CI	.46	.07	—	
	Lower 95% CI	.38	–.03	—	
	Pearson’s R	.12***	.46***	.23***	—
4. MHC	Higher 95% CI	.17	.50	.27	—
	Lower 95% CI	.07	.42	.18	—

Note. *** p < .001

Factorial invariance (*Table 3*) by gender was evaluated through multigroup confirmatory factor analysis with WLSMV estimation. The configural model showed acceptable fit (χ^2^(22) = 85.91; CFI= .990; TLI = .980; RMSEA = .080 [.062–.098]; SRMR = .024), supporting structural equivalence. The metric model showed equivalent indices (χ^2^(28) = 89.64; CFI= .990; TLI = .985; RMSEA = .069 [.054–.086]; SRMR = .028), with ΔCFI= .000 and ΔRMSEA = −.011, supporting metric invariance. The scalar model, with equal thresholds, maintained excellent fit (χ^2^(62) = 109.36; CFI= .992; TLI = .995; RMSEA = .041 [.028–.053]; SRMR = .025), and changes relative to the metric model were minimal (ΔCFI= +.002; ΔRMSEA = −.028), confirming scalar invariance. Finally, the strict model, with additional equality of residual variances, presented an equally adequate fit (χ^2^(65) = 109.42; CFI= .993; TLI = .995; RMSEA = .039 [.026–.051]; SRMR = .025), with ΔCFI= +.001 and ΔRMSEA = −.002, which confirms strict invariance by gender.

**Table 3:** Summary of Fit Indices for the Measurement Invariance Models of the Relational Self-Esteem Scale across Gender Groups

**Model**	**χ^2^(df)**	**CFI**	**TLI**	**RMSEA [IC90%]**	**SRMR**	**ΔCFI**	**ΔRMSEA**
Configural	85.91 (22)	.990	.980	.080 [.062–.098]	.024	—	—
Metric	89.64 (28)	.990	.985	.069 [.054–.086]	.028	.000	−.011
Scalar	109.36 (62)	.992	.995	.041 [.028–.053]	.025	+.002	−.028
Strict	109.42 (65)	.993	.995	.039 [.026–.051]	.025	+.001	−.002

*Note. χ^2^(df) = chi-square goodness; CFI= Comparative Fit Index; TLI = Tucker–Lewis Index; RMSEA [90% CI] = Root Mean Square Error of Approximation with 90% confidence interval; SRMR = Standardized Root Mean Square Residual; ΔCFI, ΔRMSEA = change in CFI/RMSEA relative to the less constrained model.*

## Discussion

The present study aimed to analyze the psychometric properties of the Relational Self-Esteem Scale (RSE) in a large sample of the Ecuadorian population. Taken together, the results support the reliability, validity, and one-dimensional structure of the instrument, confirming its suitability for evaluating self-esteem based on interpersonal relationships in Latin American contexts. The internal consistency indices were excellent, indicating high homogeneity among items and adequate representativeness of the construct. These values exceed the criteria recommended by Nunnally and Bernstein (1994) and are comparable to findings reported in previous studies with Asian populations (Du et al., 2012; Du et al., 2017; Li et al., 2022), where the RSE showed reliabilities between .88 and .93. The high item–total correlation and the stability of ω coefficients if an item is removed reinforce the idea that all items contribute significantly to measuring the same latent factor.

The exploratory factor analysis revealed a one-factor structure, supported by an eigenvalue of 3.26 that explained 47.8% of the total variance. The KMO and Bartlett’s tests confirmed the suitability of the data, and all factor loadings were above .40, showing clear internal coherence. These results support the theoretical conception of relational self-esteem as a one-dimensional construct centered on the perception of being valued and accepted by others (Cross et al., 2000; Du et al., 2017).

The confirmatory factor analysis using the WLSMV estimator corroborated this one-factor model, showing excellent fit indices. The factor loadings were statistically significant and of high magnitude, indicating that the items adequately reflect the latent dimension. These results are consistent with the original proposal (Du et al., 2012), but contrast with the bifactor model in a rural community sample (Du et al., 2016).

Regarding convergent validity, the average variance extracted (AVE) was .49, close to the recommended threshold of .50 (Fornell & Larcker, 1981), which indicates adequate convergence of items on the construct. The correlation with personal self-esteem, in line with Du et al. (2016), and with collective self-esteem confirms its theoretical coherence as a component of self-evaluation nurtured by social interactions and a sense of belonging. In addition, a positive relationship with psychological well-being (MHC) was found, in the expected direction. These results support that self-esteem based on positive relationships is associated with greater well-being and lower psychological symptomatology, which is consistent with previous evidence on the protective role of relational self-esteem against emotional distress (Du et al., 2017; Neff & McGehee, 2010).

Regarding factorial invariance by gender, multigroup analyses demonstrated configural, metric, scalar, and strict invariance, with minimal differences between models (ΔCFI≤ .002; ΔRMSEA ≤ .015). This indicates that the scale measures the same construct with the same structure and meaning for men and women, allowing valid comparisons between groups. This finding is particularly relevant, since the literature has pointed out possible gender differences in the internalization of relational self-esteem (Cross & Madson, 1997), but the current results suggest that the construct operates equivalently in both genders within the Ecuadorian context.

Taken together, the results confirm the Relational Self-Esteem Scale as a reliable, valid, and gender-invariant measure for evaluating self-esteem linked to interpersonal relationships. Its statistical adequacy, together with its brevity and ease of application, make it a useful tool for research on well-being, identity, and mental health in Latin American populations.

## Conclusion

The results support the reliability, validity, and one-dimensional structure of the Relational Self-Esteem Scale in the Ecuadorian population, confirming its factorial equivalence by gender. The scale constitutes a psychometrically solid tool for the study of the relational self, providing evidence on the importance of meaningful relationships as a source of personal evaluation in Latin American contexts.

## Limitations

Despite the robustness of the findings, the study presents some limitations. First, the sample was non-probabilistic and predominantly urban and young, which limits generalization to other age groups or rural contexts. Future studies should replicate these analyses with representative samples and longitudinal designs, as well as explore cultural invariance in other Latin American countries. In addition, it would be pertinent to examine the relationship of the RSE with indicators of social support, relational identity, and interpersonal stress, thereby expanding its external validity.

Taken toge ther, these findings underscore the importance of considering the relational dimension of the self as an essential component of well-being. In interdependent cultural contexts such as Latin American ones, where family and community relationships may be axes of identity and emotional support, relational self-esteem can constitute a strategic psychosocial resource for the design of public policies, mental health programs, and educational actions centered on the collective construction of well-being.

## Figures and Tables

**Table 1:** Original and Spanish versions of the Relational Self-Esteem Scale (RSE) Items

**Item**	**English Version (Du et al., 2012)**	**Spanish Adapted Version**
1	I am a worthy member of my circle of friends.	Soy un miembro digno de mi círculo de amigos.
2	In general, I’m glad to be a member of my circle of friends.	En general, me alegro de ser miembro de mi círculo de amigos.
3	I feel I have much to offer to my family.	Siento que puedo tener mucho que ofrecer a mi familia.
4	I am proud of my family.	Estoy orgullosa/o de mi familia.
5	I can help my friends a lot.	Puedo ayudar mucho a mis amigas/os.
6	Overall, my circle of friends is considered to be good by others.	En general, los demás consideran que mi círculo de amigos es bueno.
7	I think my family is proud of me.	Creo que mi familia está orgullosa/o de mi.

## Appendix

* Activities of Meta Platforms Inc. (Facebook and Instagram), and the X Corp. (as the successor of Twitter Inc.), are prohibited in the Russian Federation.
